# Multimodal Ultrasound for Assessment of Renal Fibrosis in Biopsy-Proven Patients with Chronic Kidney Disease

**DOI:** 10.1055/a-2559-7743

**Published:** 2025-03-31

**Authors:** Xinyue Huang, Tianhong Wei, Jie Li, Letian Xu, Yangshuo Tang, Jin-Tang Liao, Bo Zhang

**Affiliations:** 1 159374 Department of Ultrasound Imaging, Xiangya Hospital Central South University, Changsha, China; 2 196534 Department of Ultrasound, Nanchang University Second Affiliated Hospital, Nanchang, China

**Keywords:** Chronic kidney disease, Renal fibrosis, Shear Wave Elastography, Multimodal ultrasound, Angio Planewave Ultrasensitive Imaging

## Abstract

**Objectives:**

To establish a discriminant function model combining clinical data and multimodal ultrasound to predict the degree of renal fibrosis in patients with chronic kidney disease (CKD) and to explore the application value of the non-invasive assessment of renal fibrosis by new ultrasound techniques.

**Methods:**

Clinical data and ultrasonography, shear wave elastography, and angio planewave ultrasensitive imaging characteristics of patients with CKD were collected. The significant indicators were screened to establish discriminant function models to distinguish the degree of renal fibrosis, and the diagnostic efficacy was evaluated.

**Results:**

The 158 patients were divided into 4 groups according to pathological results. The significant indicators among or within the 4 groups were mainly age, estimated glomerular filtration rate, serum creatinine, peak systolic velocity and resistance index of renal arteries, kidney elasticity, and arcuate artery vascular density (p<0.05). The discriminant function models exhibited good diagnostic efficiency and higher accuracy compared to any single indicator.

**Conclusion:**

The SWE elasticity value of the kidney increases with the degree of fibrosis, while AP can visualize microvascular conditions qualitatively and quantitatively. Multimodal ultrasound combined with clinical data is a non-invasive strategy for the assessment of renal fibrosis.

## Zusammenfassung

**Ziel:**

Entwicklung eines Diskriminanz-Modells, das klinische Daten und multimodalen Ultraschall kombiniert, um den Grad der Nierenfibrose bei Patienten mit chronischer Nierenerkrankung (CKD) vorherzusagen, und um den Nutzen der nicht invasiven Bewertung der Nierenfibrose mittels neuer Ultraschalltechniken zu untersuchen.

**Material und Methoden:**

Von Patienten mit CKD wurden klinische Daten und Bildgebungsmerkmale aus Ultraschall, Scherwellen-Elastografie und „Angio Planewave Ultrasensitive Imaging“ gesammelt. Die signifikanten Indikatoren wurden ausgewertet, um Diskriminanz-Modelle zur Feststellung des Grades der Nierenfibrose zu erstellen, und deren diagnostische Aussagekraft wurde bewertet.

**Ergebnisse:**

158 Patienten wurden anhand der pathologischen Befunde in 4 Gruppen eingeteilt. Die signifikanten Indikatoren zwischen den 4 Gruppen oder innerhalb von ihnen waren hauptsächlich Alter, geschätzte glomeruläre Filtrationsrate, Serumkreatinin, systolische Spitzengeschwindigkeit und Widerstandsindex der Nierenarterien, die Nierenelastizität und die Gefäßdichte der Arteria arcuata (p<0,05). Die Diskriminanz-Modelle zeigten eine gute diagnostische Aussagekraft und eine höhere Genauigkeit im Vergleich zu jedem einzelnen Indikator.

**Schlussfolgerung:**

Der SWE-Elastizitätswert der Niere steigt mit dem Grad der Fibrose, während AP mikrovaskuläre Zustände qualitativ und quantitativ darstellen kann. Der multimodale Ultraschall in Kombination mit klinischen Daten stellt eine nicht invasive Strategie zur Beurteilung der Nierenfibrose dar.

## Introduction

Chronic kidney disease (CKD) is a significant global public health concern and affects over 800 million individuals worldwide 1 . In 2013, CKD was among the top 10 causes of Disability Adjusted Life Years (DALYs) 2 . In China, CKD prevalence was estimated at 10.8%, suggesting that approximately 120 million adults are affected. The rapid increase in hypertension and diabetes incidence in China over the past 15–20 years implies that individuals may develop CKD within a decade, thus presenting an opportunity for early intervention to prevent progression to end-stage renal disease 3 .

The primary pathological process in CKD is progressive fibrosis, which is a result of sustained damage to glomeruli, renal tubules, renal vessels, or interstitium from various pathogenic factors. This damage ultimately leads to glomerulosclerosis, renal tubule interstitial inflammation, or interstitial fibrosis 4 . Thus, understanding and objectively assessing renal fibrosis are crucial for guiding treatment and prognosis.

Currently, the estimated glomerular filtration rate (eGFR) is commonly used to assess renal function impairment ( Table 1 ), but there is a lack of sensitivity for early detection. Kidney biopsy is primarily used to diagnose the underlying causes of asymptomatic haematuria and proteinuria, as well as to assess primary and secondary kidney diseases, hereditary conditions, acute kidney injury, and transplanted kidneys. It helps determine the etiology, degree of pathology, and pathological type. However, kidney biopsy is not suitable for patients with severe hypertension, significant bleeding tendencies, single kidney, or renal atrophy. As a result, some patients with CKD are unable to undergo biopsy to identify the cause or stage of their condition. While kidney biopsy remains the gold standard for diagnosing renal fibrosis, its invasiveness poses risks such as infection, bleeding and renal function injury, thus limiting its use for follow-up 5 .

**Table:** **Table 1** Classification of CKD.

Stage	Description	GFR mL/(min·1.73m ^2^ )
1	Kidney injury(+), normal	Greater than 90
2	Kidney injury(+), mildly decreased	60–89
3	Moderately decreased	30–59
4	Severely decreased	15–29
5	Kidney failure	Less than 15
**Note:** The table is from the People’s Republic of China Health Industry Standard WS/T 557–2017. Positive indicators of kidney injury include abnormal blood and urine composition or abnormal imaging.		

Morphological changes in CKD, including glomerulosclerosis, interstitial fibrosis, and tubular atrophy, lead to alterations such as increased parenchymal stiffness and changes in renal vessels. Ultrasonography (US) is the preferred imaging modality for kidney disease screening. However, differentiating between diseased and healthy kidneys using conventional ultrasound can be challenging. Recent advancements, such as shear wave elastography (SWE) and angio planewave ultrasensitive imaging (AP), offer valuable insight into tissue hardness and microvessel imaging, thereby enhancing disease diagnosis 6 .

SWE has shown significant progress in diagnosing liver and breast diseases by assessing tissue hardness 9 . AP, a novel ultrasound technology mainly used for superficial organs, has limited research but shows promise based on similar technologies like superb microvascular imaging (SMI), which can detect subtle changes in small blood vessels and predict fibrosis severity 11 .

To date, no studies have explored whether clinical data and US, SWE, and AP characteristics can determine the degree of renal fibrosis. This study aims to develop a discriminant function model based on clinical data and US, SWE, and AP features, in order to establish a noninvasive strategy for the early detection and quantification of renal fibrosis in CKD patients.

## Materials and Methods

### Patients

Our study continuously enrolled 253 patients with CKD in our hospital from April 2022 to February 2023. The study was approved by our hospital’s ethics research board, and written informed consent was obtained from all patients.

Inclusion criteria: 1) Patients with proteinuria, haematuria, or elevated creatinine; 2) all patients underwent renal conventional ultrasound, SWE, and AP; 3) renal biopsy with a definite pathological diagnosis was completed within 7 days after ultrasound examination.

Exclusion criteria: 1) The ultrasound examination image is not clear and the data are not satisfactory, because the kidney position is too deep; 2) kidney with occupying lesions, renal calculi, hydronephrosis; 3) history of renal surgery or puncture.

### Ultrasonography procedures

We performed the ultrasound scans using the Supersonic Aixplorer ultrasound system (Supersonic, France) with a SC6–1U convex array probe (3–5MHz) for conventional ultrasound and SWE and an SL10–2 linear array probe (7–10MHz) for AP software. The scans were conducted with the patient in the horizontal and left lateral position, focusing on the right kidney. Measurements of renal size, flow velocity, and resistance index (RI) were obtained for the main renal artery (MRA), segmental renal artery (SRA) and interlobar renal artery (IRA). SWE and AP measurements were performed 3–4 times in the cortical area of the inferior pole of the kidney, which corresponded to the location of renal biopsy and were acquired with patients holding their breath. A region of interest with a diameter of 5.0mm was placed in the cortical area to measure elasticity, with a standard deviation (SD) of less than 1.5 as a quality control threshold. The flow velocity and RI of the arteriae arciformes renis (AAR) in the renal cortex were measured using an overall flow map showing the flow from the SRA to the AAR branch. All examinations were conducted by a qualified radiologist who was blinded to the patient data.

**Fig. 1:**
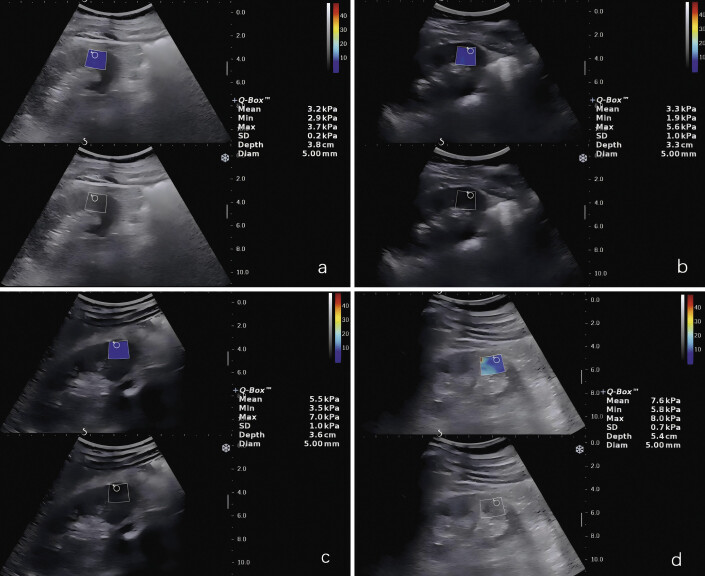
Example of kidney elasticity under SWE: **a** In patients without fibrosis, the mean elasticity was 3.2 kPa. **b** In patients with mild fibrosis, the mean elasticity was 3.3 kPa. **c** In patients with moderate fibrosis, the mean elasticity was 5.5 kPa. **d** In patients with severe fibrosis, the mean elasticity was 7.6 kPa.

**Fig. 2:**
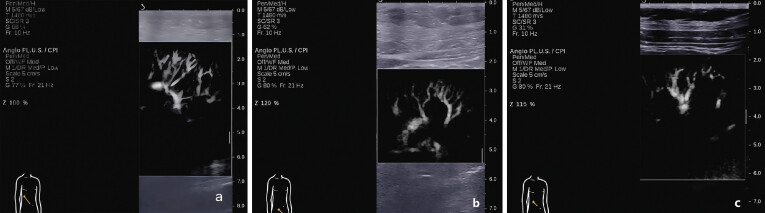
Example of kidney image and blood vessel display on AP: **a** In patients with vascular density grade 1, the arcuate vessels appear clear and fine. **b** In patients with vascular density grade 2, the arcuate vessels were sparse and less clear. **c** In patients with vascular density grade 3, the arcuate vessels showed significant reduction and sparseness.

The measurement methods and an example of results are shown in Fig. 1 (SWE) and Fig. 2 (AP), respectively.

### Kidney biopsy and histopathologic examination

Ultrasound-guided renal biopsies were performed at the inferior pole of the right kidney within 7 days. Semi-quantitative assessment was performed based on pathological findings. Referring to the scoring criteria introduced by Li et al. ( Table 2 ) 12 , histological scores were divided into 4 categories based on glomerular sclerosis, tubulointerstitial injury, and vascular sclerosis: non-fibrosis (8 points), mild fibrosis (9 points), moderate fibrosis (10–18 points), and severe fibrosis (≥19 points).

**Table:** **Table 2** Renal pathology scoring criteria.

Grades	Glomerulus (3–12)			Renal parenchymal injury (3–9)			Blood vessel (2~6)	
	Proliferation	Segmental lesions	Sclerosis	Renal interstitial inflammatory cell infiltration	Renal interstitial fibrosis	Renal tubule atrophy	Blood vessel wall thickens	Arterial hyaline qualitative change
1	<25%	≤10%	≤10%	≤25%	≤25%	≤25%	≤25%	≤25%
2	25–50%	10–25%	10–25%	25–50%	25–50%	25–50%	25–50%	25–50%
3	>50–75%	>25–50%	>25–50%	≥50%	≥50%	≥50%	≥50%	≥50%
4	≥75%	≥50%	≥50%	NA	NA	NA	NA	NA
NA=not applicable								

### Statistical analyses

Statistical tests were performed using SPSS version 26.0 (IBM). Continuous variables with normal distribution were expressed as means (SD), while those with non-normal distribution were expressed as medians (interquartile ranges). Categorical variables were presented as counts and frequencies. Univariate analysis of variance was used to compare measurement data with a normal distribution among groups, and Tamhane’s T2 test was used for comparison of measurement data with a non-normal distribution among groups. Categorical variables were compared between groups using the Chi-square tests or Fisher’s exact tests. Fisher’s discriminant analysis was employed for multifactor analysis and internal testing. Receiver operating characteristic (ROC) curves were plotted to calculate the area under the curve (AUC), sensitivity, specificity, and accuracy. A *p* -value of less than 0.05 was considered statistically significant.

## Results

### Participant basic information

**Fig. 3:**
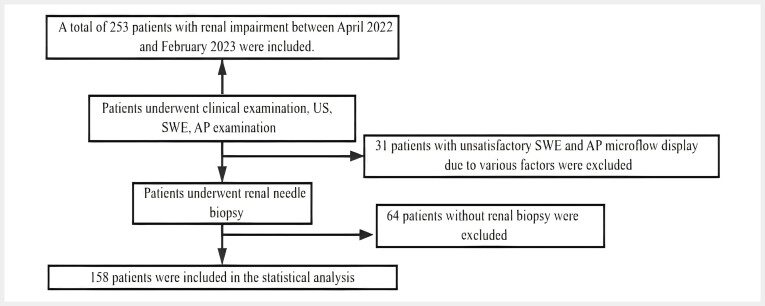
Flowchart of enrolled patients.

A total of 158 patients meeting the inclusion and exclusion criteria were ultimately included in the analysis. 64 patients did not undergo renal biopsy because of severe hypertension and significant bleeding tendencies ( Fig. 3 ). The degree of fibrosis in patients with different pathological types is shown in Table 3 , and the overall cases of tubulointerstitial diseases are rare. According to the pathological diagnosis, 158 patients were divided into 4 groups: 27 cases (17.1%) with no fibrosis, 39 cases (24.7%) with mild fibrosis, 67 cases (42.4%) with moderate fibrosis, and 25 cases (15.8%) with severe fibrosis. The study consisted of 80 males (50.6%) and 78 females (49.4%), ranging in age from 18 to 73 years old.

**Table:** **Table 3** The degree of fibrosis in patients with different pathological types.

Pathological classification	Number	No fibrosis	Mild impairment	Moderate impairment	Severe impairment
**Primary glomerulonephritis**	107				
IgA nephropathy	47	6	11	26	4
Minimal change primary glomerulonephritis	4	4	0	0	0
Focal segmental glomerulosclerosis	5	0	1	4	0
Membranous nephropathy	21	2	10	7	2
Mesangial proliferative glomerulonephritis	17	6	5	5	1
Crescent body glomerulonephritis	5	0	0	2	3
Intravascular proliferative glomerulonephritis	3	2	1	0	0
IgM nephropathy	1	1	0	0	0
Proliferative sclerosis and sclerotic nephritis	4	0	0	1	3
**Secondary glomerulonephritis**	46				
Lupus nephritis	19	1	5	10	3
Purpura nephritis	5	2	3	0	0
Diabetic nephropathy	2	0	0	2	0
Monoclonal immunoglobulin deposition disease	3	0	1	1	1
Amyloidosis of kidney	2	0	2	0	0
ANCA-associated vasculitis renal damage	7	0	0	2	5
Hypertensive renal damage	8	0	0	6	2
**Tubulointerstitial disease**	5				
Granulomatous interstitial nephritis	2	0	0	1	1
Acute renal tubular injury	3	3	0	0	0

Univariate analysis of variance was performed to compare the general data among the 4 groups or pairwise comparisons were performed within the groups. The results showed statistical differences in these indicators, including: 1) Clinical data: age, eGFR, serum creatinine (Scr), uric acid, systolic blood pressure (SBP), diastolic blood pressure (DBP); 2) conventional US: peak systolic velocity (PSV), end diastolic velocity (EDV), and resistance index (RI) of MRA, SRA, IRA; 3) SWE: kidney elasticity in left recumbent position (LPKE) and kidney elasticity in prone position (PPKE); 4) AP: PSV, EDV, and RI of AAR, renal arcuate artery vascular density (RAAVD) ( *P* <0.05, Table 4 ).

**Table:** **Table 4** Patient characteristics at baseline.

Characteristic	No fibrosis	Mild impairment	Moderate impairment	Severe impairment	*p* -value
Age (years)	30.5±17.6	410±17.2 ^a^	44.8±14.5 ^a^	59.4±12.3 ^ab^	<0.001
Gender (male/female)	13/14	16/23	38/29	13/12	0.36
BMI (kg/m ^2^ )	21.6±8.1	23.0±4.3	23.9±3.5	25.0±3.5	0.166
eGFR(mL/min/1.73 ^2^ )	123.2 (17.0)	111.1 (26.6)	76(59.5) ^ab^	6.5(5.8) ^abc^	<0.001
SCr (µmol/L)	51.0 (16.0)	56.5 (31.0)	91.0(70.5) ^ab^	684(373.5) ^abc^	<0.001
uric acid (µmol/L)	358.2±134.7	335.7±106.8	393.5±101.1 ^b^	451.8±137.9 ^b^	0.025
24h Ualb (mg/d)	3.51 (6.66)	0.89 (3.99)	3.3 (3.73)	0.9 (5.31)	0.329
UACR	3.41 (6.08)	0.77 (5.23)	2.71 (4.05)	1.09 (3.26)	0.316
SBP (mmHg)	118.8±19.9	122.6±20.3	136.7±25.3 ^ab^	152.8±24.8 ^ab^	0.001
DBP (mmHg)	74.8±9.0	78.2±12.4	86.8±15.5 ^ab^	81.6±18.2	0.001
Renal length (mm)	103.0±2.0	104.1±1.6	105.6±1.3	97.6±4.6	0.344
Renal volume (cm ^3^ )	115.8±8.1	128.8±6.7	139.6±5.3	117.7±18.9	0.092
MRA PSV (cm/s)	109.5±35.8	91.5±22.2 ^a^	84.2±22.9 ^a^	74.1±28.3 ^a^	<0.001
MRA EDV (cm/s)	37.2±12.5	31.1±9.4 ^a^	28.6±9.1 ^a^	13.1±4.5 ^a^	<0.001
MRA RI	0.65±0.09	0.66±0.07	0.65±0.09	0.82±0.03 ^abc^	0.001
SRA PSV (cm/s)	52.8±22.5	43.7±13.8 ^a^	41.2±15.2 ^a^	25.3±7.5 ^ac^	0.002
SRA EDV (cm/s)	20.6±8.3	17.0±5.8 ^a^	15.4±4.5 ^a^	6.1±2.2 ^ac^	<0.001
RSA RI	0.60±0.07	0.61±0.08	0.6±0.1	0.8±0.1 ^ab^	0.187
IRA PSV (cm/s)	24.0±5.6	25.1±9.1	21.5±7.2	15.6±8.0 ^abc^	0.016
IRA EDV (cm/s)	10.5±2.4	10.6±3.9	9.0±2.9 ^ab^	5.0±2.5 ^abc^	<0.001
IRA RI	0.55±0.07	0.58±0.07	0.57±0.08	0.67±0.12 ^abc^	0.027
AAR PSV (cm/s)	8.3±2.8	8.2±2.5	8.1±2.8	8.9±3.2	0.917
AAR EDV (cm/s)	3.9±1.2	3.9±1.3	3.5±1.2	2.4±1.4 ^abc^	0.055
AAR RI	0.51±0.09	0.52±0.08	0.56±0.08 ^ab^	0.72±0.1 ^abc^	<0.001
ROI LSD (mm)	4.3±1.4	4.3±1.5	4.7±1.5	6.8±2.1 ^abc^	0.005
LPKE (kPa)	3.8±0.7	4.1±0.9	4.4±0.9 ^a^	4.4±1.2	0.028
ROI PPD (mm)	4.5±1.1	4.2±1.1	4.3±1.2 ^ab^	5.6±1.3 ^abc^	0.074
PPKE (kPa)	3.5±0.9	3.9±0.7	4.3±0.8 ^ab^	4.2±0.9	<0.001
RAAVD					<0.001
Grade 1	24 _α_	34 _α_	22 _β_	0 _β_	
Grade 2	3 _α_	8 _α_	35 _β_	4 _αβ_	
Grade 3	0 _α_	0 _α_	10 _α_	21 _β_	
**Notes:**^a^*p* <0.05 vs. no fibrosis; ^b^ *p* <0.05 vs. mild impairment; ^c^ *p* <0.05 vs. moderate impairment. α and β represent subsets of different categories. At *p* =0.05 level, there is no statistical difference between groups with the same letter, but there is a statistical difference between groups with different letters. **Abbreviations:** BMI (body mass index); eGFR (estimated glomerular filtration rate); SCr (serum creatinine); 24h Ualb (24h urinary protein); UACR (urinary albumin-to-creatinine ratio); PSV (peak systolic velocity); EDV (end diastolic velocity); RI (resistance index); MRA (main renal artery); SRA (segmental renal artery); IRA (arteriae interlobulares renis); AAR (arteriae arciformes renis); LPKE (kidney elasticity in left recumbent position); PPKE (kidney elasticity in prone position); RAAVD (renal arcuate artery vascular density)					

### Fisher’s discriminant function model

According to Table 4 , the statistically significant indicators for comparison were selected to create predictive variables between the fibrosis (mild, moderate, severe) and non-fibrosis groups, including age, eGFR, SBP, DBP, PSV, and EDV of MRA, PSV and EDV of SRA, PSV and EDV of IRA, EDV and RI of AAR, and the LPKE. The fibrosis group was assigned a value of 0, and the non-fibrosis group was assigned a value of 1. The data matrix of non-fibrosis and fibrosis was analyzed using SPSS software to obtain Fisher’s discriminant models (F1) related to clinical data, US, SWE, and AP.

The Fisher discriminant function for non-fibrosis is:

*Z1* =0.296 × age + 0.208 × eGFR + 0.060 × SBP + 0.390 × DBP + 0.335 × MRA PSV 0.295 × MRA EDV 0.632 × SRA PSV + 1.889 × SRA EDV + 0.339 × IRA EDV + 97.204 × AAR RI + 1.737 × LPKE 81.759

The Fisher discriminant function for fibrosis is:

*Z2* =0.330 × age + 0.194 × eGFR + 0.053 × SBP + 0.429 × DBP + 0.303 × MRA PSV 0.275 × MRA EDV 0.592 × SRA PSV + 1.694 × SRA EDV + 0.480 × IRA EDV + 94.301 × AAR RI + 1.932 × LPKE 80.301

Substituting each variable value from the original data into the aforementioned discriminant function, the prediction groups were calculated, and their values were compared. The overall accuracy of the discriminant function in the non-fibrosis group was found to be 0.774.

The statistically significant indicators for comparison were selected to create predictive variables between the moderate to severe fibrosis group and the mild fibrosis group, including age, eGFR, uric acid, SBP, DBP, MRA PSV, MRA EDV, SRA PSV, IRA PSV, IRA EDV, LPKE,PPKE and RAAVD. The mild fibrosis group was assigned a value of 1, and the moderate-severe fibrosis group was assigned a value of 2. SPSS software was utilized to analyze the data matrix of mild fibrosis and moderate-severe fibrosis, resulting in Fisher’s discriminant model F2.

The Fisher discriminant function for mild fibrosis is:

*Z3* =0.596 × age + 0.292 × eGFR + 0.065 × uric acid + 0.094 × SBP + 0.491 × DBP + 0.333 × MRA PSV 0.107 × MRA EDV + 0.059 × SRA PSV 0.619 × IRA PSV + 1.105 × IRA EDV + 1.562 × LPKE + 2.335 × PPKE + 5.524 × RAAVD 88.044

The Fisher discriminant function for moderate to severe fibrosis is:

*Z4* =0.617 × age + 0.261 × eGFR + 0.075 × uric acid + 0.061 × SBP + 0.594 × DBP + 0.329 × MRA PSV 0.042 × MRA EDV + 0.106 × SRA PSV 0.700 × IRA PSV + 0.976 × IRA EDV + 1.765 × LPKE + 2.391 × PPKE + 6.327 × RAAVD 96.425

By substituting the data into the aforementioned discriminant function, the comprehensive accuracy of the discriminant function in predicting the mild fibrosis group was found to be 0.847.

**Fig. 4:**
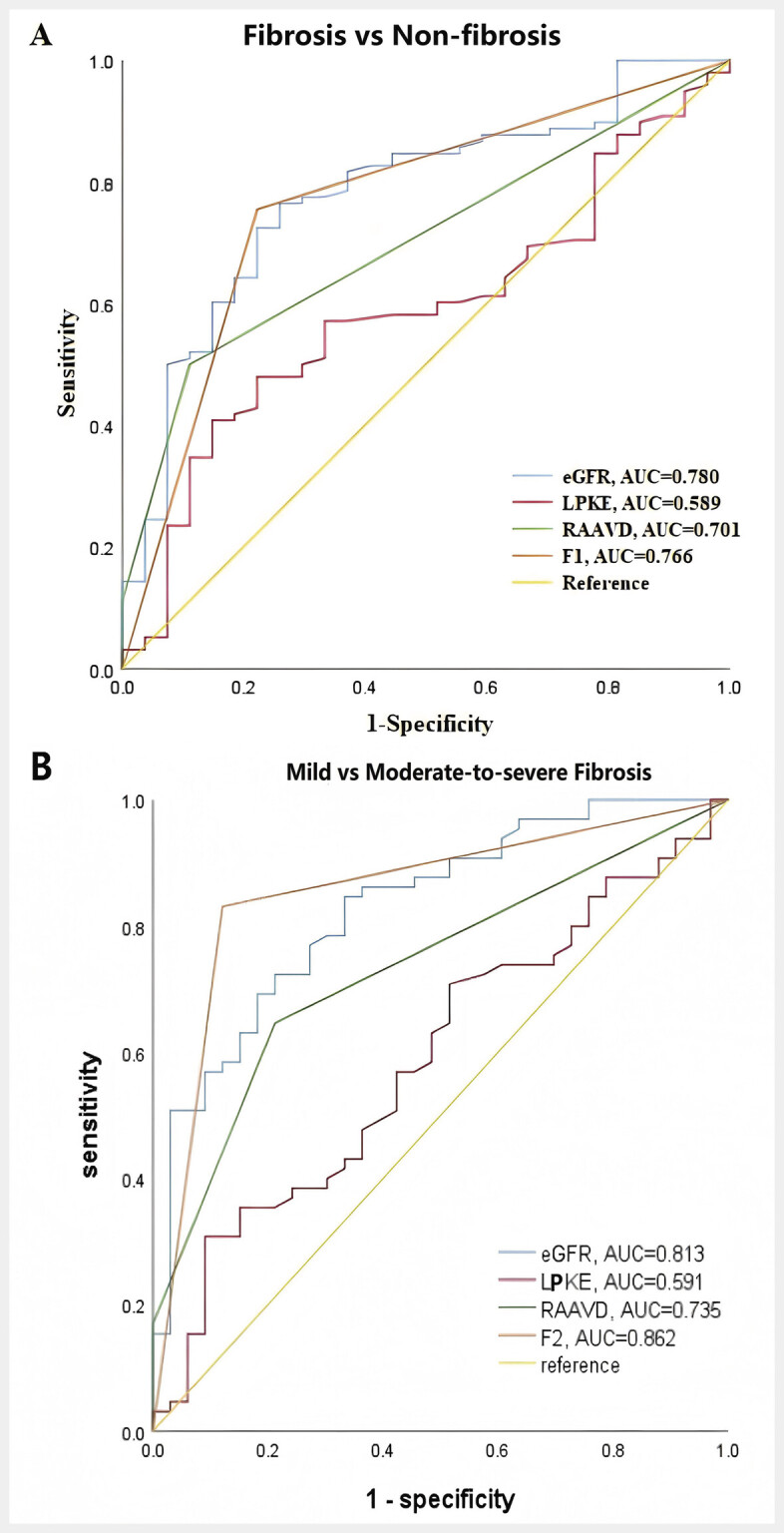
ROC curve of single index and discriminant function for the diagnosis of renal fibrosis: **A** Renal fibrosis vs. no-fibrosis. **B** Renal mild fibrosis vs. moderate-to-severe fibrosis.

The ROC curve was used ( Fig. 4 ) to assess the diagnostic ability of eGFR, LPKE, RAAVD, and the discriminant function for renal fibrosis, as well as for mild and moderate-severe fibrosis ( Table 5 ).

**Table:** **Table 5** Diagnostic efficacy of renal fibrosis.

	Mild fibrosis					Moderate-severe fibrosis				
Index	eGFR	eGFR	LSKE	RAAVD	F1	eGFR	eGFR	LSKE	RAAVD	F2
Cut-off	114.2	90	4.46	/	/	98.3	60	5.76	/	/
Sensitivity	0.741	0.926	0.571	0.5	0.755	0.816	0.974	0.909	0.788	0.939
Specificity	0.764	0.464	0.667	0.889	0.778	0.681	0.375	0.308	0.646	0.785
Accuracy	0.752	0.547	0.562	0.584	0.774	0.727	0.573	0.509	0.694	0.847
p-value	<0.001	/	0.059	<0.001	<0.001	<0.001	/	0.144	<0.001	<0.001
AUC [95%CI]	0.780 [0.638–0.856]	/	0.589 [0.476–0.701]	0.701 [0.603–0.799]	0.766 [0.663–0.870]	0.813 [0.733–0.893]	/	0.591 [0.474–0.708]	0.735 [0.635–0.835]	0.862 [0.785–0.939]
**Notes:** At present, a clinical eGFR greater than 90 refers to stage CKD1. When the eGFR cut-off value is 90, the sensitivity is 0.926, the specificity is 0.464, and theaccuracy is 0.547; an eGFR value between 60 and 90 is clinically defined as stage CKD2. If 90 is the cut-off value, the sensitivity is 0.842, the specificity is 0.625, and the accuracy is 0.691. If 60 is the cut-off value, the sensitivity is 0.974, the specificity is 0.375, and the accuracy is 0.573.										

## Discussions

Current clinical drugs are primarily utilized to alleviate symptoms and delay the progression of kidney disease. However, the evidence regarding the potential slowdown, inhibition, or even reversal of fibrosis is still in the preliminary stage (preclinical stage) 13 . Therefore, early diagnosis and intervention play a crucial role in the treatment of CKD and in impeding its progression. To our knowledge, previous studies have utilized SWE and a similar method called supersonic molecular imaging (SMI) separately to evaluate chronic liver fibrosis 9 , indicating their importance in liver fibrosis evaluation.

In the study, we compared clinical data and US, SWE and AP indicators across different patient groups with renal fibrosis. A discriminant function model was developed and validated using these variables to predict the extent of renal fibrosis. It is essential to emphasize that additional research and validation are necessary to fully ascertain the effectiveness of SWE and AP in evaluating renal fibrosis. Early diagnosis and intervention are crucial for the effective management of CKD and for slowing its progression.

Our analysis included various clinical features to identify early indicators of renal fibrosis. Among these, the eGFR is particularly significant for early detection. eGFR was calculated using the MDRD formula (Modification of Diet in Renal Disease), which primarily relies on Scr. Although eGFR is widely used in clinical practice, its sensitivity, specificity, and accuracy in distinguishing between individuals with and without fibrosis, as well as between those with mild versus moderate-to-severe fibrosis were limited. The Ualb and urinary albumin-to-creatinine ratio (UACR) did not show statistical significance with respect to determining the severity of renal fibrosis. In our Fisher’s discriminant models, eGFR achieved an AUC of 0.747 (95%-CI: 0.638–0.856) for discriminating between fibrosis and non-fibrosis, and 0.813 (95%-CI: 0.733–0.893) for distinguishing mild from moderate-to-severe fibrosis. These results indicate that the discriminant function model, which includes clinical features, outperformed the model based on clinical features alone. Thus, a discriminant function model incorporating clinical data could be valuable for the non-invasive monitoring of renal fibrosis severity.

Ultrasound is a commonly used tool for screening and monitoring kidney diseases, allowing assessment of kidney size, cortical echogenicity, arterial spectra at various levels, and overall blood flow. Despite its high specificity, ultrasound has limited sensitivity in screening chronic kidney disease. Our study found that certain parameters, including PSV, EDV, and RI of MRA, RSA, and IRA, were statistically significant for determining the degree of renal fibrosis. Notably, PSV and EDV across all arteries decreased with increasing severity of renal fibrosis. These findings are consistent with those studies, which observed a strong correlation between changes in systolic and diastolic blood flow and renal cortical fibrosis. PSV and EDV serve as semiquantitative indicators of renal blood flow, reflecting the dilation of renal arterioles. Arterial interstitial fibrosis limits the dilation of arteries and arterioles, resulting in reduced compliance, increased resistance, and diminished perfusion of renal vessels 15 . Consequently, we included all relevant ultrasound variables from CKD patients in the discriminant function model.

According to the American Kidney Database 17 , approximately one-third of patients with end-stage renal disease (ESRD) have associated renal vascular disease. This condition primarily manifests as renal aortic and branch stenosis or thromboembolic microangiopathies. Atherosclerosis is the leading cause of 70–90% of cases of renal vessel stenosis. Persistent inflammation also contributes to CKD by promoting ongoing fibrosis repair, vasoconstriction, and mesangial contraction, which ultimately reduces renal perfusion. While increasing systemic blood pressure is necessary to improve renal perfusion, hypertension can exacerbate kidney injury. Research has shown that ischemia can damage the kidneys and stimulate fibrosis formation. Hypoxia-inducible factor (HIF), released by ischemic cells, promotes angiogenesis and provides protection. However, under chronic hypoxic conditions, HIF has been shown to promote renal fibrosis. These findings indicate that renal fibrosis, driven by various factors, can lead to insufficient renal blood perfusion. Conversely, stenosis and occlusion of renal vessels can exacerbate renal fibrosis. Consequently, renal blood flow can serve as an indicator of the severity of renal fibrosis.

In our study, AP technology was used to visualize the arcuate artery, the smallest branch of the renal artery. We categorized vascular density into 3 levels, based on the density of the arcuate artery displayed by AP. Our results demonstrated that arcuate artery vascular density was statistically significant for determining the severity of renal fibrosis. As fibrosis progressed, microvascular occlusion became more pronounced, resulting in reduced arcuate artery density and higher vascular density grades. Immunohistochemistry revealed a significant reduction in microvessels within the intracortical area by 59% and medulla by 49% in CKD patients compared to the control group, confirming the presence of fibrosis and capillary thinning in CKD patients 18 . Our findings are consistent with these results.

SWE is a non-invasive technique used to assess tissue hardness. In our study, elastic values increased across the non-fibrotic, mild fibrosis, moderate fibrosis, and severe fibrosis groups, although these differences were not statistically significant. Only the elastic values for the severe fibrosis group were significantly higher than those of the non-fibrosis, mild fibrosis, and moderate fibrosis groups. Some studies suggest that insufficient blood perfusion and poor capillary filling in the early stages of CKD can lead to decreased elasticity, which may mask the increased elasticity caused by renal fibrosis. Renal elasticity is influenced not only by the degree of renal fibrosis but also by factors such as the voiding status, hemodynamic status, general atherosclerotic status, intra-renal blood volume, and renal depth 19 . Since SWE requires a sufficient amplitude to produce detectable tissue displacement via ultrasound, attenuation increases with frequency, making the elasticity value dependent on tissue depth.

Additionally, we evaluated the accuracy of elasticity measurements taken in different body positions. The values measured in the left and prone positions did not significantly differ with respect to detecting non-fibrosis. However, the left lateral position was more effective than the prone position in differentiating non-fibrosis from mild renal fibrosis, which is consistent with the findings of Yang 20 . Therefore, combining AP imaging with kidney elasticity values improves the diagnostic performance of the discriminant function model.

Fisher’s discriminant analysis was conducted on all clinical data, including US, SWE, and AP, which were statistically significant in distinguishing non-fibrotic from mild renal fibrosis. The results indicated that the discriminant function achieved the highest AUC values for differentiating between the presence and absence of fibrosis (Z1, Z2) and between mild fibrosis and moderate-to-severe fibrosis (Z3, Z4). The discriminant model, as shown in Fig. 4 , can be utilized in clinical practice for detecting renal fibrosis, particularly for patients who cannot undergo a renal biopsy during follow-up. Establishing an ultrasound-based model will support clinical practice and may aid in predicting the severity of renal fibrosis in the future.

However, the study has several limitations. Firstly, it is a single-center study with a patient cohort limited to those with pathological results from renal puncture. This results in a small number of severe cases and a relatively high proportion of primary glomerulonephritis, potentially introducing a selection bias. Secondly, the elastic parameters were fixed, and the kidney depth was not individualized for each patient. Future research should categorize kidney depth into 3 groups: < 2cm, 2–4cm, and > 4cm, and use varying frequencies of elastic parameters (high, medium, and low) to minimize the impact of kidney depth. Thirdly, the study’s sample size is limited, and the discriminant function was validated only internally. External validation with an independent dataset is recommended. Finally, ongoing monitoring and validation of patients are necessary for future studies.

### Conclusion

SWE and AP techniques significantly enhance diagnostic efficiency for early renal fibrosis. Integrating clinical data with US, SWE, and AP results allows the discriminant function to markedly improve the diagnostic accuracy for early renal fibrosis. This non-invasive approach demonstrates high precision for assessing the severity of renal fibrosis.
